# Supplementary material for: Comparative Analysis of mRNA, microRNA of Transcriptome, and Proteomics on CIK Cells Responses to GCRV and Aeromonas hydrophila
Source: Int J Mol Sci. 2024 Jun 11;25(12):6438. doi: 10.3390/ijms25126438 (PMC11204273; doi:10.3390/ijms25126438)
Supplement: Supplementary file 1 [file ijms-25-06438-s001.zip › Figure S1.pdf]

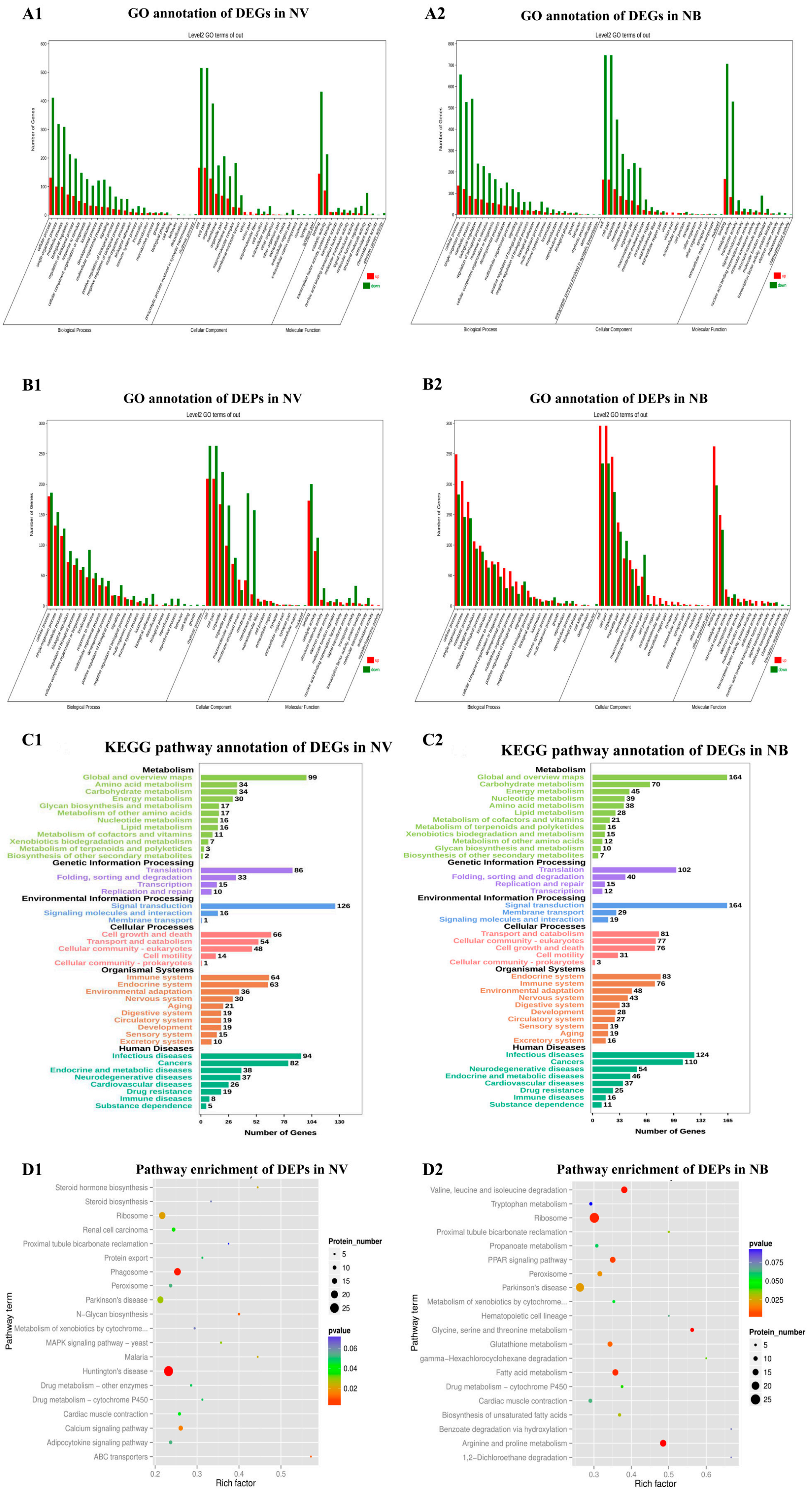
A1
GO annotation of DEGs in NV
Level2 GO terms of out
Number of Genes
up
down
Biological Process
Cellular Component
Molecular Function
A2
GO annotation of DEGs in NB
Level2 GO terms of out
Number of Genes
up
down
Biological Process
Cellular Component
Molecular Function
B1
GO annotation of DEPs in NV
Level2 GO terms of out
Number of Genes
up
down
Biological Process
Cellular Component
Molecular Function
B2
GO annotation of DEPs in NB
Level2 GO terms of out
Number of Genes
up
down
Biological Process
Cellular Component
Molecular Function
C1
KEGG pathway annotation of DEGs in NV
Metabolism
Global and overview maps 99
Amino acid metabolism 34
Carbohydrate metabolism 34
Energy metabolism 30
Glycan biosynthesis and metabolism 17
Metabolism of other amino acids 17
Nucleotide metabolism 16
Lipid metabolism 16
Metabolism of cofactors and vitamins 11
Xenobiotics biodegradation and metabolism 7
Metabolism of terpenoids and polyketides 3
Biosynthesis of other secondary metabolites 2
Genetic Information Processing
Translation 86
Folding, sorting and degradation 33
Transcription 15
Replication and repair 10
Environmental Information Processing
Signal transduction 126
Signaling molecules and interaction 16
Membrane transport 1
Cellular Processes
Cell growth and death 66
Transport and catabolism 54
Cellular community - eukaryotes 48
Cell motility 14
Cellular community - prokaryotes 1
Organismal Systems
Immune system 64
Endocrine system 63
Environmental adaptation 36
Nervous system 30
Aging 21
Digestive system 19
Circulatory system 19
Development 19
Sensory system 15
Excretory system 10
Human Diseases
Infectious diseases 94
Cancers 82
Endocrine and metabolic diseases 38
Neurodegenerative diseases 37
Cardiovascular diseases 26
Drug resistance 19
Immune diseases 8
Substance dependence 5
0 26 52 78 104 130
Number of Genes
C2
KEGG pathway annotation of DEGs in NB
Metabolism
Global and overview maps 164
Carbohydrate metabolism 70
Energy metabolism 45
Nucleotide metabolism 39
Amino acid metabolism 38
Lipid metabolism 28
Metabolism of cofactors and vitamins 21
Metabolism of terpenoids and polyketides 16
Xenobiotics biodegradation and metabolism 15
Metabolism of other amino acids 12
Glycan biosynthesis and metabolism 10
Biosynthesis of other secondary metabolites 7
Genetic Information Processing
Translation 102
Folding, sorting and degradation 40
Replication and repair 15
Transcription 12
Environmental Information Processing
Signal transduction 164
Membrane transport 29
Signaling molecules and interaction 19
Cellular Processes
Transport and catabolism 81
Cellular community - eukaryotes 77
Cell growth and death 76
Cell motility 31
Cellular community - prokaryotes 3
Organismal Systems
Endocrine system 83
Immune system 76
Environmental adaptation 48
Nervous system 43
Digestive system 33
Development 28
Circulatory system 27
Sensory system 19
Aging 19
Excretory system 16
Human Diseases
Infectious diseases 124
Cancers 110
Neurodegenerative diseases 54
Endocrine and metabolic diseases 46
Cardiovascular diseases 37
Drug resistance 25
Immune diseases 16
Substance dependence 11
0 33 66 99 132 165
Number of Genes
D1
Pathway enrichment of DEPs in NV
Pathway term
Steroid hormone biosynthesis
Steroid biosynthesis
Ribosome
Renal cell carcinoma
Proximal tubule bicarbonate reclamation
Protein export
Phagosome
Peroxisome
Parkinson's disease
N-Glycan biosynthesis
Metabolism of xenobiotics by cytochrome...
MAPK signaling pathway – yeast
Malaria
Huntington's disease
Drug metabolism – other enzymes
Drug metabolism – cytochrome P450
Cardiac muscle contraction
Calcium signaling pathway
Adipocytokine signaling pathway
ABC transporters
0.2 0.3 0.4 0.5
Rich factor
Protein_number
5
10
15
20
25
pvalue
0.06
0.04
0.02
D2
Pathway enrichment of DEPs in NB
Pathway term
Valine, leucine and isoleucine degradation
Tryptophan metabolism
Ribosome
Proximal tubule bicarbonate reclamation
Propanoate metabolism
PPAR signaling pathway
Peroxisome
Parkinson's disease
Metabolism of xenobiotics by cytochrome...
Hematopoietic cell lineage
Glycine, serine and threonine metabolism
Glutathione metabolism
gamma–Hexachlorocyclohexane degradation
Fatty acid metabolism
Drug metabolism – cytochrome P450
Cardiac muscle contraction
Biosynthesis of unsaturated fatty acids
Benzoate degradation via hydroxylation
Arginine and proline metabolism
1,2–Dichloroethane degradation
0.3 0.4 0.5 0.6
Rich factor
pvalue
0.075
0.050
0.025
Protein_number
5
10
15
20
25

Figure S1. The function annotation analysis of DEGs and DEPs in both groups. (A, B) GO enrichment of DEGs and DEPs in NV and NB groups. (A1) and (A2) represent GO annotations of DEGs in NV and NB groups, respectively. (B1) and (B2) represent GO annotations of DEPs in NV and NB groups, respectively. Red represents up-regulated DEGs and green represents down-regulated DEGs. (C) KEGG enrichment analysis of DEGs in NV and NB groups. KEGG pathway enrichment of DEGs between NV and N (C1), NB and N (C2). (D) KEGG pathway annotation of DEPs. Top 20 statistics of KEGG pathway in NV and NB group. This scatter plot-rich factor was the ratio of DEP numbers annotated in this KEGG pathway term to all protein numbers annotated in this KEGG pathway term. KEGG pathway enrichment of DEPs between NV and N (D1), NB and N (D2).
