# Supplementary material for: Comparative Analysis of mRNA, microRNA of Transcriptome, and Proteomics on CIK Cells Responses to GCRV and Aeromonas hydrophila
Source: Int J Mol Sci. 2024 Jun 11;25(12):6438. doi: 10.3390/ijms25126438 (PMC11204273; doi:10.3390/ijms25126438)
Supplement: Supplementary file 1 [file ijms-25-06438-s001.zip › Table S1.pdf]

Table S1. Preliminary transcriptome analysis

sample	Raw Reads	Raw Base	Clean Reads	Clean Base	mapped	Q20%	Q30%	GC%
N	110000000	16.5G	108231404	16.23G	71504193	97.67	94.69	47.47
NV	110000000	16.5G	108128554	16.22G	77243475	97.28	93.99	47.27
NB	110000000	16.5G	108002520	16.20G	73744247	97.44	94.31	47.47
