# Supplementary material for: Comparative Analysis of mRNA, microRNA of Transcriptome, and Proteomics on CIK Cells Responses to GCRV and Aeromonas hydrophila
Source: Int J Mol Sci. 2024 Jun 11;25(12):6438. doi: 10.3390/ijms25126438 (PMC11204273; doi:10.3390/ijms25126438)
Supplement: Supplementary file 1 [file ijms-25-06438-s001.zip › Table S2.pdf]

Table S2. Preliminary of small RNA-seq

| miRNA-seq | N | NV | NB |
|---|---|---|---|
| Raw reads | 10013600 | 9860023 | 12016812 |
| Valid reads | 9,093,062, | 9,164,280 | 10,901,955 |
| Unique sequence | 237,975 | 173,912 | 95,825 |
| rRNA | 28095 | 13279 | 28476 |
| tRNA | 70557 | 64114 | 50594 |
| snoRNA | 2160 | 1887 | 2736 |
| snRNA | 1741 | 1441 | 2699 |
| Other Rfam RNA | 4183 | 2164 | 2742 |
