# Supplementary material for: Comparative Analysis of mRNA, microRNA of Transcriptome, and Proteomics on CIK Cells Responses to GCRV and Aeromonas hydrophila
Source: Int J Mol Sci. 2024 Jun 11;25(12):6438. doi: 10.3390/ijms25126438 (PMC11204273; doi:10.3390/ijms25126438)
Supplement: Supplementary file 1 [file ijms-25-06438-s001.zip › Table S3.pdf]

Table S3. Primer list of selected DEGs in RT-qPCR verification experiments

	F Primer (5'→3')	R Primer (5'→3')
GAPDH	GTTACAAGGGAGAAGTTCACCAT	CCGGTAGACTCGACTACATACAG
Syvn1	TGACTGGAGCCAAGAAAC	CATACGACAGGTGGGACA
edf1	AAGCCATTCCCAATAACC	GCTGCCCAATATCCTTTC
CDO1	CACCGCAACACTGTCAAG	CTCTTCCCACTGTCCTTTG
BMS1	GAGGAGATGCGATGGACT	CAGCCTTTGCTTTGTGAA
ZYX	ACCATCGCCGTTTCTCTC	ATCCTCACATCGGTAGCAC
UGDH	GAAAGCCAAAGCATAAGA	AAACAGAGGAGTCCGTAG
csnk1g1	CTCAGGTCTACTATTTCGGG	AATGGCGATCATCAGGAC
CSNK2A1	CAGACTACGACATTCGGTTC	GTTCATGGTCGATCATTACG
HDAC7	AGTCGTCTCCTGCTTCCAC	TCAGCATTTGGGAGTCGTA
Ube2ib	AAGTCGCCTCGCACAAGA	AACAAGCCTCCCTCCCAC
Gtf2a1	TCAGCCTCAAGCACAGCC	CTCGCCACCATCTTTCTC
cpt2	GCCGCCATAAACCACAAC	CCATAGCCCACTCCGAAA
dlg1	GCAAGAAGAGGGTGGAGA	TCGCTGGCGTTAGATGTT
PTPN1	CAACCCGACGACCCAATC	AGGTGCGGTAACAGACATA

Primer list of selected DEMs in RT-qPCR verification experiments

	Stem-loop Primer(F)	Stem-loop Primer(R)	Reverse transcription primer(5'-3')
miR-223_R+1	GCGCGCGGCAG GCAAA	AGTGCAGGGTCCG AGGTATT	GTCGTATCCAGTGCAGGGTCCGAGG TATTCGCACTGGATACGACTGGGGT
PC-3p-4398_72	CGCGCGCCGGG AGCCC	AGTGCAGGGTCCG AGGTATT	GTCGTATCCAGTGCAGGGTCCGAGG TATTCGCACTGGATACGACGGCCGC
mir-24-5-p5	GCGCGCGGCCG CGGCG	AGTGCAGGGTCCG AGGTATT	GTCGTATCCAGTGCAGGGTCCGAGG TATTCGCACTGGATACGACCTGTTT
PC-3p-18726_11	GCGACGCAGAA GGGCC	AGTGCAGGGTCCG AGGTATT	GTCGTATCCAGTGCAGGGTCCGAGG TATTCGCACTGGATACGACGGCGTC
PC-3p-4801_64	GCGCGCAGAGG ACACCAG	AGTGCAGGTCCGA GGTATT	GTCGTATCCAGTGCAGGGTCCGAGG TATTCGCACTGGATACGACGGTGTC
miR-9226_L-4_1ss22GT	GCGCGCGCGGC CCGCG	AGTGCAGGGTCCG AGGTATT	GTCGTATCCAGTGCAGGGTCCGAGG TATTCGCACTGGATACGACGGCGCC

Primer list of HDAC7-3'UTR and VDAC2-3'UTR

	Primer(F)	Primer(R)
HDAC7-3'UTR	GCTAGCCTCGAGTCTAGA	CTGCAGGTCGACTCTAGA
	GGTCTTTTTACTTGTCTG	CACTTCAATAGTTCGCTT
VDAC2-3'UTR	GCTAGCCTCGAGTCTAGA	CTGCAGGTCGACTCTAGA
	ACTCCAACAGCAACCAGCAG	CAGGGAATCAGGGTCATAAT
