# Supplementary material for: Comparative Analysis of mRNA, microRNA of Transcriptome, and Proteomics on CIK Cells Responses to GCRV and Aeromonas hydrophila
Source: Int J Mol Sci. 2024 Jun 11;25(12):6438. doi: 10.3390/ijms25126438 (PMC11204273; doi:10.3390/ijms25126438)
Supplement: Supplementary file 1 [file ijms-25-06438-s001.zip › Table S4.pdf]

Table S4. The GO term enrichment of specific proteins of NV group and NB group

Category	GO_Term	GO_ID	Protein Num
NB-BP	transcription, DNA-dependent	GO:0006351	19
	transport	GO:0006810	17
	translation	GO:0006412	16
	regulation of transcription, DNA-dependent	GO:0006355	16
NB-MF	metal ion binding	GO:0046872	34
	protein binding	GO:0005515	34
	GTP binding	GO:0005525	29
	ATP binding	GO:0005524	29
NB-CC	cytoplasm	GO:0005737	83
	nucleus	GO:0005634	79
	cytosol	GO:0005829	44
NV-BP	protein transport	GO:0015031	20
	intracellular protein transport	GO:0006886	15
	cell division	GO:0051301	14
NV-MF	protein binding	GO:0005515	36
	ATP binding	GO:0005524	34
	GTP binding	GO:0005525	23
NV-CC	cytoplasm	GO:0005737	64
	integral to membrane	GO:0016021	60
	nucleus	GO:0005634	54
