# Supplementary material for: Comparative Analysis of mRNA, microRNA of Transcriptome, and Proteomics on CIK Cells Responses to GCRV and Aeromonas hydrophila
Source: Int J Mol Sci. 2024 Jun 11;25(12):6438. doi: 10.3390/ijms25126438 (PMC11204273; doi:10.3390/ijms25126438)
Supplement: Supplementary file 1 [file ijms-25-06438-s001.zip › Table S5.pdf]

Table S5. The names corresponding to the Pathway ID

| NV Sample | |
|---|---|
| Pathway_ID | Pathway Name |
| ko04620 | Toll-like receptor signaling pathway |
| ko04012 | ErbB signaling pathway |
| ko04062 | Chemokine signaling pathway |
| ko04621 | NOD-like receptor signaling pathway |
| ko00010 | Glycolysis / Gluconeogenesis |
| ko00053 | Ascorbate and aldarate metabolism |
| ko00040 | Pentose and glucuronate interconversions |
| Ko03010 | Ribosome |
| NB Sample | |
| ko00053 | Ascorbate and aldarate metabolism |
| ko00010 | Glycolysis / Gluconeogenesis |
| ko00020 | Citrate cycle (TCA cycle) |
| ko00030 | Pentose phosphate pathwa |
| ko00040 | Pentose and glucuronate interconversions |
| ko00051 | Fructose and mannose metabolism |
| ko02010 | ABC transporters |
| ko03070 | Bacterial secretion system |
| ko04145 | Phagosome |
| ko04142 | Lysosome |
| ko04144 | Endocytosis |
| ko04612 | Antigen processing and presentation |
| ko04621 | NOD-like receptor signaling pathway |
