# Supplementary material for: Comparative Analysis of mRNA, microRNA of Transcriptome, and Proteomics on CIK Cells Responses to GCRV and Aeromonas hydrophila
Source: Int J Mol Sci. 2024 Jun 11;25(12):6438. doi: 10.3390/ijms25126438 (PMC11204273; doi:10.3390/ijms25126438)
Supplement: Supplementary file 1 [file ijms-25-06438-s001.zip › Table S6.pdf]

Table S6. Statistics of KEGG enrichment of DEPs in NV and NB group

Sample	Pathway	Pathway-ID	Specific-genes Num
NV	Endocytosis	ko04144	15
	MAPK signaling pathway)、	ko04010	12
	Chemokine signaling pathway	ko04062	11
	Phagosome	ko04145	11
	Tight junction	ko04530	11
	Focal adhesion	ko04510	10
	Oxidative phosphorylation	ko00190	8
	Purine metabolism	ko00230	8
	Neurotrophin signaling pathway	ko04722	8
	Regulation of actin cytoskeleton	ko04810	8
NB	Regulation of actin cytoskeleton	ko04810	20
	Focal adhesion	ko04510	16
	Valine, leucine and isoleucine degradation	ko00280	15
	Tight junction	ko04530	15
	Fatty acid metabolism	ko00071	14
	Arginine and proline metabolism	ko00330	13
	Adherens junction	ko04520	13
	Propanoate metabolism	ko00640	12
	Oxidative phosphorylation	ko00190	11
	Purine metabolism	ko00230	11
