# Supplementary material for: Comparative Analysis of mRNA, microRNA of Transcriptome, and Proteomics on CIK Cells Responses to GCRV and Aeromonas hydrophila
Source: Int J Mol Sci. 2024 Jun 11;25(12):6438. doi: 10.3390/ijms25126438 (PMC11204273; doi:10.3390/ijms25126438)
Supplement: Supplementary file 1 [file ijms-25-06438-s001.zip › Table S7.pdf]

Table S7. Statistics of immune pathways of DEGs and DEPs in NV and NB group

	Genes/Proteins Num in Immune pathway	Main Immune pathway-ID
DEGs in NV group	57	ko04612, ko04660, ko04662, ko04621, ko04622, ko04620, ko04630, ko04666, ko04062
DEGs in NB group	84	ko04612, ko04660, ko04670, ko04630, ko04666, ko04620, ko04621, ko04662, ko04062
DEPs in NV group	41	ko04062, ko04666, ko04662, ko04620, ko04660, ko04670
DEPs in NB group	45	ko04062, ko04670, ko04666, ko04660, ko04662, ko04650
