# Supplementary figures and images for: Comparative Analysis of mRNA, microRNA of Transcriptome, and Proteomics on CIK Cells Responses to GCRV and Aeromonas hydrophila
Source: Int J Mol Sci. 2024 Jun 11;25(12):6438. doi: 10.3390/ijms25126438 (PMC11204273; doi:10.3390/ijms25126438)

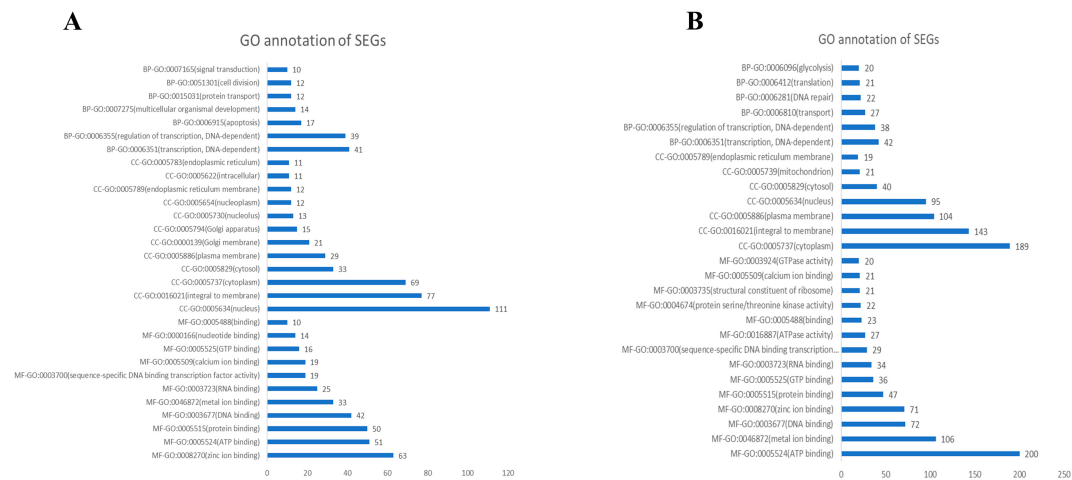

**Figure S2.** The top 30 GO terms of specific genes in NV and NB group.

Supplement: Supplementary file 1 [file ijms-25-06438-s001.zip › Figure S2.pdf]
